# Nineteenth Century Medicine*An Address delivered before the Alumni Association of the Medical Department of the University of Georgia, April 1, 1897.

**Published:** 1897-05

**Authors:** William Whatley Battey

**Affiliations:** Augusta, Ga., President of the Alumni Association


### NINETEENTH CENTURY MEDICINE.*

BY WILLIAM WHATLEY BATTEY, M.D., AUGUSTA, GA.,
President of the Alumni Association.

I am deeply grateful to you for having selected me to preside over the present meeting of the Alumni Association of the Medical College of Georgia; a college whose graduates have been scattered all over our Southern country; some of whom may be found in

*An Address delivered before the Alumni Association of the Medical Department of the University of Georgia, April 1, 1897.

almost every State in the Union; and even on other shores and in other climes many of them have been peers of any in our profession, and some of whom have been teachers of note in some of the best medical colleges in our country.

Our grand old Alma Mater, from its inception in 1828 to the present time, has been first and foremost in progressiveness and in the advocacy of the higher and more perfect education of its pupils, and has kept well abreast with all the discoveries in our science. And to-day I say it, and I believe with truth, that in all the advantages it offers both in didactics and in clinics, medical and surgical, it has no superiors; not only in our Southern cities, but even in the larger cities North and West, where the greatest geniuses, talents, and facilities are supposed to be found, and nowhere is there a more able or proficient corps of professors. Gentlemen, I am proud of the venerable old institution for the good she has done and is still doing, and I am sure you all have the same feeling of pride and affection for our Alma Mater.

I hope, fellow alumni, that we will continue to do everything in our power to foster and sustain our dear old college; and let us endeavor to fill her halls and class-rooms with bright, intelligent young men and help to make her the largest, the best, the grandest, and most complete medical university in this, our Southern country.

Now, gentlemen, in paying this brief and humble tribute to our college, it occurs to me as not out of place to make a few remarks on the progress of that science that she has fostered, encouraged and taught so well, and for the advancement of which she has done so much. I will, therefore, make a few remarks on "Nineteenth Century Medicine."

Since the beginning of our college, over sixty years ago, medical science has made many advances and recorded numerous triumphs, more so, perhaps, than in all her previous history, and I think we can say without exaggerating that her grandest achievements, in the line of progress and discovery, have been made within the last thirty years. It is true, in the centuries past, many illustrious men, as Hippocrates, Boerhaave, Vesalius, Sydenham, Harvey, Jenner, Broussais, Hunter, Cooper, Trousseau, and numerous others have done much by their discoveries in advancing the progress of medicine in all its branches, and helped to open the way to further progress in our science. Yet with the exception of Harvey's grand discovery of and demonstration of the circulation, and Jenner's great boon of vaccination for the prevention of small-pox, that dread plague, worse than Atilla, "the Scourge of God," claiming its thousands and tens of thousands in epidemic after epidemic yet, I say, with the exception of the discoveries of these two illustrious men, the principal achievements, the greatest triumphs, and the most numerous discoveries in medicine in all its branches have been made during this present century, and largely in the latter half of it. And medicine, which previously was largely empirical, is rapidly becoming more of an exact science; and as we look back upon its progress from time to time, we cannot help but wonder and admire its expansive, comprehensive, and startling growth, and the great benefits it is bestowing on humanity in alleviating its sufferings, and developing, improving, and making more perfect its species.

Let us for a few moments revive some of the advances and discoveries made in the healing art since the commencement of the present century: First, I mention the discovery of anesthesia, that blessed alleviator and destroyer of pain, by the means of which the operating room of the surgeon has been converted from a "Dante's Inferno" almost to "the Elysian fields," or to "the shores of Lethe," and from a "Procrustean bed" to one of roses. And with what a feeling of pride can we turn to the first discoverer of this priceless boon to suffering humanity and claim him as one of our own, "a Georgia Cracker," and unless I am misinformed, who once trod these venerated halls as a student, one of the children of our Alma Mater, though not a graduate of our institution, viz., Dr. Crawford W. Long. All honor to him and his co-discoverers, Drs. Morton and Wells; and well may all future generations "arise and call them blessed."

The next greatest discovery was antisepsis, and the parent of the greater principle still of asepsis; and though some idea of antisepsis had probably been suggested long before the time of Sir Joseph Lister, yet he was the first to put it to the practical test and bring it prominently before the profession. Crowned heads have heaped honors upon his head, and greater honors have been bestowed on him in his recognition by his professional brethren, as one of the great benefactors of the human race. And though antisepsis, as first practiced by Lister, has been abandoned by himself and others in the profession, it led up to asepsis; and by the elaboration and perfection of this latter principle almost all things are possible to the skillful surgeon in his operations and explorations of the living human anatomy. And there is hardly any former *terra incognita* within the human frame, that the bold and skillful surgeon, with clean hands and clean instruments and appliances, will not explore with impunity, and relieve diseased conditions or remove affected tissues or organs, thus restoring to life and health patients who before the time of asepsis were as surely condemned to death as the criminal on the scaffold.

How many wonderful discoveries have been brought to light and possibilities opened up through the medium of that marvelous little instrument, the microscope, with its improvements in lenses and technique, overthrowing by its revelations many ancient and time honored theories of diseases and their causes. All honor to Pasteur and Davaigne, who, scarcely thirty years ago, succeeded in identifying the anthrax bacillus, showing its real nature, its pathological significance, and to the former for his discovery of the microbe of hydrophobia. He, I believe, is justly entitled to the honor of being called the father of pathological bacteriology; not only for his discovery and elucidation of the pathological significance of these micro-organisms mentioned, but for the specific methods for their prevention, and cure of the diseases of which they were the cause, by the injection of attenuated viruses, thus being the originator or suggestor of antitoxins, serum therapy and the like.

Following on the same line, numerous other bacteriologists have made similar discoveries of disease germs; also the bacteria of cell resistance in the demonstration of the action of the phogocytes and leucocytes on pathogenic bacteria.

Following Pasteur and Davaigne comes the illustrious Koch, of Germany, in his discoveries of bacillus tuberculosis, the typhoid bacillus conjointly with Eberth, and the coma bacillus of cholera.

And we are all familiar with his experiments with the attenuated virus of the bacillus tuberculosis for the cure of that dread disease, which was to revolutionize its therapeusis and drive it out of the universe; but it proved to be a two-edged sword, and has been pretty well abandoned by himself and his followers, more honored now in the breach than in the observance of its use.

I should have given credit to Davaigne as being the first to have demonstrated the pathological significance of bacteria in disease, in his experiments with the anthrax bacillus; but to Pasteur belongs the credit of having first used the attenuated virus of the anthrax bacillus for the treatment of the disease caused by this micro-organism. Following Davaigne, Obermeier, in 1872, discovered the germ of relapsing fever. Hansen, in 1879, discovered the bacillus of leprosy, and in the same year Neisser the gonococcus of gonorrhea. In 1880 Eberth and Koch, independently of each other, discovered the typhoid bacillus. In 1880 Pasteur gave to the profession the result of his investigation of the treatment of fowl cholera and anthrax by the attenuated virus of these diseases, and thus opened a new chapter in the method of treatment of infectious diseases. In 1880 our distinguished physician, Sternberg, discovered the pneumococcus of pneumonia. In 1882 Koch again came before the public with his discovery of the bacillus tuberculosis before mentioned. Also, in 1882 Löffler and Schutz discovered the micro-organism of glanders. In 1884 the coma bacillus of cholera was brought to the light by the indefatigable Koch. The bacillus of diphtheria was discovered by Löffler in 1884, and also the micro-organisms of pus by Rosenbach, and the bacillus of tetanus by Nicolaier. Lastly, in 1892, the bacillus of influenza by Pfeiffer. Another discovery, which I was about to omit mentioning, that of Laveran, of the plasmodium malariæ.

Of the many experiments made by bacteriologists in the way of prevention and cure of diseases caused by the various bacteria by the inoculation of attenuated viruses, antitoxins, serums, and the like, nothing has so interested the profession or been so universally tried as the treatment of diphtheria by antitoxin, as formulated by Behring, Roux, and others; and an immense mass of statistics have been brought to bear, both for and against its usefulness. The subject is still *sub judice*, and in a few years, like Koch's tuberculin treatment, may be as a thing of the past or a tale that is told.

In regard to serum therapy, the *rationale* of its action has undergone several different explanations by its experimenters, and its efficacy as a cure or prevention against germ diseases has not been decided, but statistics show much in its favor, and its laborious investigators deserve the highest praise for their experimental work on this line, which may bring forth good fruit.

I should not forget to mention, also, the animal alkaloids, ptomaines and leucomaines; and we point with pride to the investigations of our distinguished American physicians, Vaughan and Novey, who have added so much to our knowledge of these subjects.

It may be well to give a passing notice to the use of animal extracts which, by a sort of selection, are supposed to be useful in the treatment of similar organs in a diseased condition in the human economy, the value of which, as a rule, seems problematical, with the exception of the use of the thyroid extract, for diseased conditions produced by the atrophy or absence of this gland in the human subject, the efficacy of which has been reasoned out by physiological experiments and deductions.

On this line, in speaking of the medical profession, Phelps says: "It is evident that the present disposition to direct medical and surgical investigation straight to its ultimate end, the cure of disease, while it has grown out of the mental attitude characteristic of the time, has been greatly strengthened by the fact that conditions have been made favorable by the work previously accomplished: physiologists and microscopists, clinicians and pathologists, have so thoroughly established premises that the time has been ripe for conclusions. They have followed convergent lines, which have at last so far merged as to form a broadened course, in which persistence has disclosed the possibilities which we have seen so largely realized. The story of the bacteria, which it has taken nearly two centuries to learn, and to which the study of many sciences has been tributary, their history, classification, their life and death, and their pathogenic potentialities have become well known, when contemporary investigators were enabled to write the final chapter which reveals in these atoms the proximate cause of disease, and in some instances to touch the limit which has been set to human knowledge in this direction by the discovery of specific methods for their cure. The identification of individual micro-organisms still goes on, and efforts to make the poison its own antidote are still unwearied."

What, before the last twenty-five years, was known of cerebral localization? Now the various motor and sensory centers, affecting almost any nerve or group of nerves in different portions of the body, can be mapped out according to symptons, and the surgeon can confidently cut down upon the tissues as previously indicated on his chart.

And now comes another anesthesia discovery, and, in minor surgery, a rival of anesthesia by inhalation, where the patient can, smilingly and in serenity, and without pain, become an interested spectator of the operation, and thus the cocaine of Kohler has become quite an addition to our resources.

The disagreeable, bulky and nauseous remedies of a generation or two ago have been largely relegated to the past, and are now administered in concentrated forms, as the condensed extracts, alkaloids and the like. All the genius of the confectioner's art is often brought to bear to render them more palatable; and when such arts fail or the stomach is rebelious, we shoot them under the integument by means of that useful little instrument, the hypodermic syringe; and we get quicker and more positive results than by the old way of the alimentary canal. And were it not for this useful little instrument where would be our serum therapy and the use of the various antitoxins?

The mechanical arts and the laws of physics themselves are constantly brought to bear to assist us in the treatment of diseases. The thunderbolts of Jove himself, electricity, that powerful and all-pervading force in nature, the knowledge of which is yet in its infancy, is made an obedient servant to our beck and call, potent as a therapeutical and surgical means in diagnosis and treatment of diseases.

The cathode rays, which apparently disregard the laws of physics in penetrating opaque bodies like glass, is, I think, destined to play an important part in medicine and surgery, and be a valuable adjunct in diagnosticating obscure pathological conditions within the human anatomy. And useful as it is now in detecting foreign bodies by its wonderful penetrating searchlight in man's interior, its powers are far from being fully developed, and much greater results may be looked for from it in the future.

The introduction of the use of the tracheal tube by O'Dwyer has added another valuable instrument to our armamentarium, and helps to rob croup, diphtheria, and stenosis of the tracheal canal of its terrors and to diminish their mortality.

Though the achievements and triumphs, gentlemen, of medicine for the last fifty or a hundred years have been marvelous in the extreme and of incalculable benefit to the human race, yet the greatest good, the most astonishing results, have been realized in the line of preventive medicine; and the general trend of the profession is in that direction. I do not refer so much to the means of rendering the human economy immune from diseases by injection of serums, attenuated viruses, and the like (though much is being attempted in that line, and, like vaccination for small-pox, such means, if successful, may be classed under the head of "preventive medicine"), but I refer to preventive medicine which not only takes that in, but goes beyond to that knowledge of the laws of sanitation and hygiene that enables us not only to destroy the disease-producing germs, but to prevent the propagation of the germs themselves, and to put men in the most favorable condition to resist or throw off these germs, with healthy surroundings. Much has been done in this line by sanitation in the last few decades, but we are far yet from realizing the ideal condition of preventive medicine. As it is they are far in advance of their time, the general public not being educated up to their ideas; men, women, children, and infants are daily stricken down in great numbers by diseases that are entirely preventable, the dear public regarding such deaths as dispensations of an all-wise Providence, to whose decrees they must bow with pious resignation. Well may we say, man's insanitation, instead of inhumanity, to man makes countless thousands mourn.

In conclusion, gentlemen, allow me to say that the triumphs of medicine, its discoveries and elucidation of means of prevention and cure of diseases, are not due alone to the great geniuses, men of talent in the profession, but to those who persevere, who learn to bring to bear the faculty of close observation and the power of careful analysis and comparison ; these are capable of adding much to the vast increments of knowledge in the profession.

